# Surgical treatment of displaced intra-articular calcaneal fractures: is bone grafting necessary?

**DOI:** 10.1007/s10195-013-0246-y

**Published:** 2013-05-14

**Authors:** A. K. Singh, K. Vinay

**Affiliations:** 1 Department of Orthopedics, Mayo Institute of Medical Sciences, C 1/157, Vishesh Khand, Gomti Nagar, Gadia, Barabanki, Uttar Pradesh 226010 India; 2 Department of Orthopedics, Institute of Medical Sciences BHU, Varanasi, India

**Keywords:** Calcaneus, Displaced intra-articular fractures, Bone graft

## Abstract

**Background:**

The aim of this retrospective study was to determine the need for bone grafting in the surgical treatment of displaced intra-articular calcaneal fractures. We reviewed 390 cases of displaced intra-articular calcaneal fractures treated with plate osteosynthesis with or without autologous iliac bone grafting, and compared outcomes and complications related to fracture stabilization.

**Materials and methods:**

Three hundred ninety patients with displaced intra-articular calcaneal fractures that were treated with plate osteosynthesis from December 2002 to December 2010 were reviewed. Two hundred two patients (group A) were treated by osteosynthesis with autologous bone grafting, and 188 patients (group B) were treated by osteosynthesis without bone grafting. One hundred eighty-one patients with an AO type 73-C1 fracture (Sanders type II), 182 patients with an AO type 73-C2 fracture (Sanders type III), and 27 patients with an AO type 73-C3 fracture (Sanders type IV) were included in this study. Bohler’s angle, the crucial angle of Gissane, and calcaneal height in the immediate postoperative period and at the 2-year follow-up were compared. Any change in the subtalar joint status was documented and analyzed. The final outcomes of all patients were evaluated by the AOFAS Ankle–Hindfoot Scale and compared in both groups.

**Results:**

The mean full weight-bearing time in group A (with bone grafting) was significantly lower (median 6.2 months, range 2.8–9.2 months) than that in group B (without bone grafting; median 9.8 months, range 6.8–12.2 months). The immediate-postoperative Bohler’s angle and that at the 2-year follow-up were significantly higher in group A. The loss of Bohler’s angle after 2 years was significantly lower in group A (mean 3.5°; 95 % CI 0.8°–6.2°) than in group B (mean 6.2°; 95 % CI 1.0°–11.2°). The average change in the crucial angle and the average change in calcaneal height were not statistically significant for either group. The infection rate in the bone grafting group was higher, though statistically insignificantly so, than in the nongrafting group (8.3 vs. 6.3 %). No significant difference was found between the groups in terms of the rates of good reduction, postoperative osteoarthritis, and subtalar fusion. Regarding the efficacy outcomes, the mean AOFAS score was lower (mean 76.4 points; 95 % CI 65.8–82.9 points) in group A than in group B (mean, 81.6 points; 95 % CI, 72.3–88.8 points), but this difference was not significant (*p* > 0.05).

**Conclusions:**

Bohler’s angle showed improved restoration and the patients returned to full weight-bearing earlier when bone grafting was used in the treatment of intra-articular calcaneal fracture. However, the functional outcomes and complication rates of both groups were similar.

## Introduction

Calcaneal fractures account for approximately 2 % of all fractures, with displaced intra-articular fractures comprising 60–75 % of these injuries [1, 2]. Displaced intra-articular fractures carry a high morbidity; 40–85 % of patients return to work within 9 months, but approximately 20 % are not able to return to work within a year, rendering intra-articular calcaneal fractures costly from a socio-economic perspective [2, 3]. Axial load in falling is the most common mechanism of calcaneal fracture. In such injuries, the lateral talar process is forced downward and breaks through the posterior articular facet, reducing Bohler’s angle [4]. Plate osteosynthesis of the intra-articular fracture is a standard treatment method. The first documented treatment of a series of calcaneal fractures with internal fixation was reported by Leriche [5] in 1922.

Ever since Lenormant first described the use of bone grafting to fill the space created after open reduction of a calcaneal fracture in 1928, this technique has maintained its popularity. Choices of bone graft have included autogenous and allogenous cancellous bone grafts, polymethylmethacrylate (PMMA), and bone substitutes. However, the need for bone grafts in the treatment of intraarticular calcaneal fracture is still controversial, and there is no strong evidence to support any functional benefits of using bone grafts [6]. Surgeons in favor of bone grafting believe that it could stimulate fracture healing, leading to early full weight-bearing; may prevent posttraumatic arthritis; and could increase mechanical strength, thus helping to prevent significant late collapse [7, 8]. Those not in favor of bone grafts have stated that the highly vascular calcaneus heals radiographically 4–8 weeks after surgery in the absence of bone grafting [9–11], that internal fixation can adequately support the articular surface, that bone grafting increases the infection rate, blood loss, and postoperative pain [12, 13], and that it is also important to consider donor site morbidity and complications involved with harvesting an autograft [14, 15]. 

The purpose of the current study was therefore to compare the outcomes and complications of two methods utilized for the surgical treatment of intra-articular calcaneal fractures. We analyzed cases of displaced intra-articular calcaneal fracture treated by open reduction and internal fixation (ORIF) with bone grafts and without bone grafts by comparing the outcomes and complications of the intra-articular calcaneal fractures in these two treatment groups. We compared the infection rates, times to full weight-bearing, reduction of the posterior facet, subtalar fusion rates, reduction of Bohler’s angle, changes in the crucial angle, changes in calcaneal height, and efficacy outcomes between the two groups.

## Materials and methods

This study was done to compare the outcomes and complications of displaced intra-articular calcaneal fractures treated with open reduction and internal fixation either with or without bone grafting. The protocol of this study was approved by the research department of the Institute of Medical Sciences, BHU. The study was authorized by the local ethical committee and was performed in accordance with the ethical standards of the 1964 Declaration of Helsinki as revised in 2000. The need for informed consent was waived by the ethical committee since the rights and interests of the patients would not be violated and their privacy and anonymity was assured by the study design. During the 8 years from December 2002 to December 2010, 454 patients with displaced intra-articular calcaneal fractures were admitted to the hospital. Medical records and X-ray films were retrieved for all of the patients who had undergone open reduction and internal fixation of the calcaneus.

**Fig. 1:**
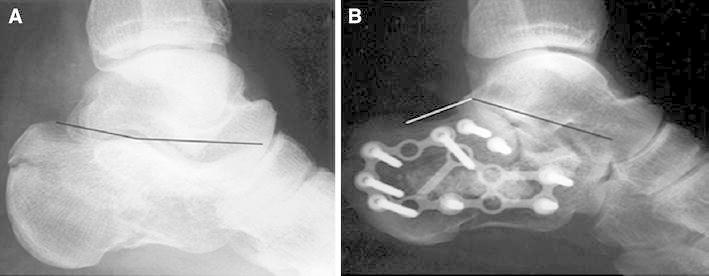
**a** Pre-op loss of Bohler’s angle in displaced intra-articular calcaneal fractures. **b** Restoration of Bohler’s angle by plate osteosynthesis

The inclusion criteria were as follows:

- Unilateral, displaced intra-articular calcaneal fractures [posterior articular facet step-off more than 2 mm, significant shortening, loss of height, and widening of the calcaneus, i.e., decreased Bohler’s and Gissane’s angles (see Fig. 1a), valgus deviation > 10°, varus deviation > 5°] of Sanders type II, III, or IV
- Age ≥ 18 years
- Records for a follow-up period of at least 2 years should be available for each case included in this study.

Three hundred ninety patients with unilateral isolated displaced intra-articular calcaneal fracture were included in this study as per the inclusion criteria. Bilateral calcaneal fractures, open fractures, and fractures in patients <18 years of age were not included in this study. Other than the demographic details, information concerning the duration of hospital stay, duration of leave, and time to full weight-bearing were collected. All the cases included in this study were divided into two groups: patients treated with bone grafting (group A) and those treated without bone grafting (group B). The average age in the bone grafting group was 40.0 (range 18–74) years and that in the nongrafting group was 41.2 (range 18–75) years. Gender proportions in the two groups were considered to be similar (*p* = 0.42). Both groups were similar in terms of the side fractured (*p* = 0.82) and the fracture type (*p* > 0.05) (Table 1).

**Table 1:** Demographic profile of study

Patient characteristics	Open reduction and internal fixation with bone grafting (group A)	Open reduction and internal fixation without bone grafting (group B)	*p* value
Sex			
Male:female	152:50	130:58	0.42
Side			
Right:left	120:82	110:78	0.82
Mean age (years)	40.0	41.2	0.64

**Fig. 2:**
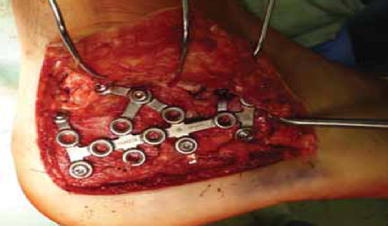
Standard L-shaped lateral approach and restoration of the posterior facet

In all cases selected, patients were operated on between the seventh and tenth day after injury (range 2–21 days, average 8.0 days), when the soft-tissue edema had decreased and the wrinkle sign was positive on hind-foot soft tissue. All of the operations were performed under general or spinal anesthesia, with the patient placed in the lateral decubitus position, using the extended lateral approach (Fig. 2). Locking plates and screws were used in all cases requiring open reduction and internal fixation. In the patients receiving a bone graft, cancellous autograft was taken from the ipsilateral iliac crest. Posterior and anterior calcaneal facet reconstruction, including the “bridging” in Gissane’s angle, Bohler’s angle (see Fig. 1b) along with calcaneal height, width, and length restoration, and no varus–valgus deviation were the main goals of open reduction.

The standard postoperative protocol was followed. Non-weight-bearing walking was started after surgery, usually on the third postoperative day. Ankle and subtalar joint mobilization exercises were started when tolerated by the patient. Progressive weight-bearing was started after 6 weeks, initially with 25 % of their weight. Full weight-bearing after 12 weeks was allowed provided that the reduced and stabilized fracture position remained unchanged and that clinicoradiological signs of bone healing (no pain, swelling, or tenderness at the fracture site clinically and no visible fracture lines in X-rays) were present at that time.

**Fig. 3:**
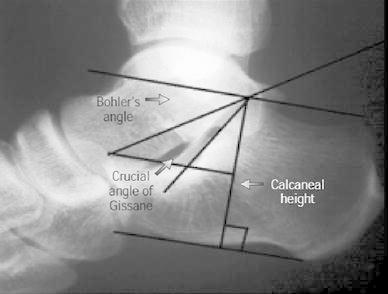
Measurement of Bohler’s angle, the crucial angle of Gissane, and the calcaneal height

The X-ray films were assessed to determine the type of fracture that had occurred. The preoperative computed tomography (CT) scans were reviewed to classify the fractures according to the Sanders classification (Table 2). The Sanders CT-scan classification is based on the articular fracture lines of the posterior articular calcaneal facet (A, lateral; B, central; C, medial), and the severity of the fracture (Sanders types I–IV) depends on the number of lines and their courses. The immediate postoperative X-rays were compared with the radiological findings at the 2-year follow-up visit. The immediate-postoperative film was taken on the second or third postoperative day. These X-rays included standard anteroposterior, true lateral, and axial views of the calcaneum. Bohler’s angle, the crucial angle, and the height of the calcaneus were measured in radiographs as shown in Fig. 3. Any change in these parameters during follow-up was documented. Serial changes in Bohler’s angle, the crucial angle, and the calcaneal height were calculated. Absolute values of the differences were used for statistical analysis. Statistical significance was taken as*p* < 0.05. The *t* test for equality was used to test for significant differences.

**Table 2:** Fracture distribution according to the Sanders classification system

Sanders classification	Group A	Group B	*p* value
Type II A	21	26	0.42
Type II B	68	56	0.36
Type II C	4	6	0.44
Type III AC	8	20	0.12
Type III AB	80	63	0.22
Type III BC	6	5	0.88
Type IV	15	12	0.68

In all cases, patient characteristics, mean follow-up time, fracture classification information based on the Sanders system, time to full weight-bearing, patient-reported outcome scores, infection, posterior facet incongruity, and subtalar joint fusion were reviewed. Sanders’ criteria was used to evaluate subtalar joint reduction, and Allmachers Arthrosis Rating Scale was employed for posttraumatic posterior facet degenerative changes (Table 3). The overall patient outcomes were summarized based on the American Orthopaedic Foot and Ankle Society Score (AOFAS) Ankle–Hindfoot Scale, with excellent defined as 90–100 points, good as 75–89 points, fair as 50–74 points, and poor as <50 points [16–18].

**Table 3:** Sanders’ criteria for subtalar joint reduction and Allmacher’s criteria for subtalar joint osteoarthrosis

Criteria for roentgenographic subtalar joint reduction [24]	Arthrosis rating scale
Anatomic, no articular incongruity whatsoever	0 Normal
Near-anatomic, <3 mm of articular incongruity or gapping between fragments	I Osteophyte only II Osteophyte with subchondral cysts and normal joint space
Approximate, 3–5 mm of articular incongruity or gapping between fragments	III Grade II + mild narrowing of the joint space
Failure, >5 mm of articular incongruity or gapping between fragments	IV Severe joint-space narrowing V Fused

## Results

The average overall duration of follow-up was 2 years. The patients of group A (with bone grafting) had an average hospital stay of 20.20 ± 5.2 days, and the group B patients (no bone grafting) had an average hospital stay of 19.02 ± 4.8 days. There was no significant difference between the two groups (*p* = 0.68).

### Functional and radiological outcomes

The mean time to full weight-bearing in group A (6.2 ± 1.7 months) was significantly lower (*p* = 0.02) than that in group B (9.8 ± 1.5 months). Bohler’s angle at the 2-year follow-up was significantly higher in group A (*p* = 0.042). The loss of Bohler’s angle after 2 years was significantly lower in group A (3.5° ± 1.4°) than in group B (mean 6.2° ± 2.5°, *p* = 0.014). The overall subtalar joint reduction was found to be satisfactory. No significant difference was found between the two groups on comparing the good reduction rate. Comparison of the immediate-postoperative radiological results and those at the 2-year follow-up showed changes in Bohler’s angle, the crucial angle, and the height of the calcaneus (Tables 4 and 5). The average change in the crucial angle was 3.8° ± 1.8° for group A and 3.6° ± 2.1° for group B. This difference was not statistically significant (*p* = 0.48). The average change in calcaneal height was 2.8 ± 1.4 mm for group A and 2.5 ± 1.2 mm for group B. Again, this difference was not statistically significant (*p* = 0.68).

**Table 4:** Preoperative and postoperative radiological assessment of both groups

Parameters	Group A			Group B		
	Average value (standard deviation)			Average value (standard deviation)		
	Type II	Type III	Type IV	Type II	Type III	Type IV
Bohler’s angle (°)						
Post-op	28.2 (6.8)	25.4 (6.1)	21.1 (7.0)	25.6 (7.4)	26.9 (8.2)	21.2 (8.4)
At 2-year follow-up	24.5 (6.9)	22.6 (6.8)	18.6 (7.2)	22.9 (7.9)	21.1 (7.8)	17.3 (7.2)
Gissane’s angle (°)						
Post-op	125.8 (8.5)	122.4 (8.0)	127.5 (8.9)	123.4 (8.8)	122.4 (10.9)	123.0 (11.2)
At 2-year follow-up	116.2 (7.6)	118.9 (7.9)	126.0 (8.1)	115.2 (9.0)	118.2 (10.2)	123.5 (9.2)
Calcaneal height (mm)						
Post-op	43.13 (3.96)	42.8 (4.12)	42.2 (4.14)	43.1 (4.14)	42.4 (4.04)	41.9 (4.12)
At 2-year follow-up	42.16 (4.10)	41.9 (4.24)	41.1 (4.28)	42.1 (4.26)	41.3 (4.24)	40.8 (4.33)

**Table 5:** Comparison of the preoperative and postoperative radiological parameters of displaced calcaneal fractures treated with plate osteosynthesis with bone grafting (group A) or without bone grafting (group B)

Radiological parameter	Group A	Group B	*p* value
	Average value ± standard deviation	Average value ± standard deviation	
Mean Bohler’s angle (°)			
Immediate post-op	28.2 ± 6.2	28.1 ± 6.3	0.52
At 2-year follow-up	25.4 ± 6.1	21.2 ± 7.2	0.042
Bohler’s angle loss	3.5 ± 1.4	6.2 ± 2.5	0.014
Mean Gissane angle (°)			
Immediate post-op	120.4 ± 8.4	118.8 ± 9.8	0.58
At 2-year follow-up	124.2 ± 7.6	122.4 ± 9.2	0.54
Angle change	3.8 ± 1.8	3.6 ± 2.1	0.48
Mean calcaneal height (mm)			
Immediate post-op	42.8 ± 3.1	42.6 ± 4.2	0.62
At 2-year follow-up	40.0 ± 3.6	40.1 ± 3.8	0.52
Height loss	2.8 ± 1.4	2.5 ± 1.2	0.68

Regarding efficacy outcomes, the mean AOFAS score was lower (76.4 ± 5.4 points) in group A than in group B (81.6 ± 4.8 points) (Table 6), but this difference was not significant (*p* > 0.82). In studies of group A, the result of treatment was excellent in 32 % of the patients, good in 39 %, fair in 24 %, and poor in 4 % of the patients. In group B, the outcome was excellent in 33 %, good in 43 %, fair in 19 %, and poor in 4 % cases. No significant difference was found on comparing both groups (*p* = 0.42).

**Table 6:** Comparison of AOFAS Ankle-Hindfoot Scores for displaced calcaneal fractures treated with plate osteosynthesis with bone grafting (group A) or without bone grafting (group B)

Sanders fracture type	Group A					Group B				
	Excellent	Good	Fair	Poor	Total	Excellent	Good	Fair	Poor	Total
Type II	32	38	22	1	93	32	40	15	1	88
Type III	30	36	25	3	94	28	37	20	3	88
Type IV	3	5	3	4	15	3	5	1	3	12
	65 (32.17 %)	79 (39.1 %)	50 (24.75 %)	8 (3.96 %)		63 (33.5 %)	82 (43.6 %)	36 (19.1 %)	7 (3.72 %)	

### Complications

In this series of 390 patients, subtalar arthrosis occurred in 50 patients (12.8 %). Twenty-six patients had superficial wound dehiscence and 25 patients had wound infection. There were no donor-site wound complications (Table 7). Among the patients with subtalar arthrosis, 27 patients were in group A (13.36 %) and 23 patients were in group B (12.7 %). This difference was not statistically significant (*p* = 0.62). The postoperative subtalar fusion rate was 3.4 % in group B and 3.2 % in group A; no significant difference was found (*p* = 0.161). The infection rate in group A was higher (6.9 %) than that in group B (5.8 %), but no statistical significant difference was found (*p* = 0.562).

**Table 7:** Complications of displaced calcaneal fractures treated with plate osteosynthesis with bone grafting (group A) or without bone grafting (group B)

Complications	Group A	Group B	*p* value
Superficial defect	12 (6.2 %)	14 (7.4 %)	0.425
Infection	14 (6.9 %)	11 (5.8 %)	0.562
Post-op osteoarthritis	27 (13.3 %)	23 (12.7) %	0.238
Subtalar fusion	7 (3.4 %)	6 (3.2 %)	0.161

## Discussion

The operative treatment of intraarticular calcaneal fractures with or without bone grafting is still a topic of debate. The practice of using a bone graft to fill the “empty core” of the calcaneus during surgery has become increasingly popular. Many authors have incorporated bone grafting into the fixation procedure, and have achieved satisfactory results [19, 20]. Some surgeons always use bone grafts [7, 16], whereas other surgeons do not use them at all [21, 22]. A nationwide survey of the Netherlands reported that, among the surgeons who operated on the ORIF group, 20 % definitely used bone grafts, 42 % used grafting when deemed necessary, and 38 % did not use bone grafts at all [23]. The objective of the present study was to compare the outcomes and complication rates of both groups (A and B) and to determine the differences between the groups in terms of final outcomes and complications.

The practice of bone grafting was not favored in early works [24–26]. Letournel [25] did not recommend the use of bone grafting, and said that although a gap is caused by cancellous crushing at the time of injury, the thalamic fragment and overall body reduction are supported by the lag screws and plate; in addition, because the medial wall is also fractured, proper filling of the space is impossible, and the speed of healing did not warrant the extra risk associated with a graft. Sanders et al. [24], Stephenson [26], and Zwipp et al. [27] achieved good results without the use of a bone graft. Lowery and Calhoun [28], in their recent extensive review of the treatment of calcaneal fracture, did not recommend the use of bone grafting. Longino [29] compared postoperative radiological and clinical results of LCP osteosynthesis with and without bone grafting and did not find any significant difference in the results obtained with or without grafting. Leung et al. [19, 20] recommended open reduction with rigid internal fixation with primary bone grafting as the preferred treatment method for calcaneal fracture.

Our results showed that both groups of patients presented similar clinical and radiological progress. Bone grafting did not hasten their return to work. The group receiving a bone graft had a slightly longer duration of hospital stay, although this was not statistically significant. This may have been due to the extra surgical wound in the iliac area and the associated pain, which may have impeded their progress in relation to mobility [7]. This systematic review showed that patients treated with bone grafts were able to return to full weight-bearing earlier. However, no significant difference was observed in the other indicators. Many studies have reported higher infection rates when treating intra-articular calcaneal fractures with bone grafts, which is one of the reasons that authors propose that bone grafts should not be used [12]. According to the results of the present study, there was no significant difference in infection rate between patients with bone grafts and those without bone grafts. Restoration of Bohler’s angle has also been associated with a better outcome [6, 30–32]. In the present work, the postoperative and 2-year follow-up radiographs were reviewed and the loss of Bohler’s angle was compared between the two groups. It was found that the mean postoperative Bohler’s angle was significantly higher and the mean loss of reduction of the angle was significantly lower in the bone-grafting group. The efficacy outcomes for the two groups were not significantly different when all of the categories were compared. In terms of the reported scores, the patients in the bone-grafting group yielded a lower mean AOFAS score. The subtalar joint reduction achieved during surgery was maintained during the long-term follow-up in both groups. This reflects the hypothesis of Loucks and Buckley [6] that surgical reduction of the subtalar joint is more important and that bone grafting does not provide any extra benefit.

The morbidity associated with bone-graft harvesting from the iliac crest has been extensively studied and reviewed. The procedure of bone-graft harvesting increases the operative time and blood loss [30]. According to Silber et al., the complication rates associated with harvesting iliac bone grafts can be as high as 40 %, and encompass acute or chronic pain, hematoma formation, scarring, nerve injury, and wound problems. Given the extra surgical morbidity and the lack of any demonstrable extra benefit associated with bone grafting, we suggest that it should not be performed during surgery for calcaneal fracture.

The primary limitation of the present study is that it is a retrospective investigation, and not all of the patient details relating to operative time, blood loss, and pain severity were available. Despite these limitations, this study provides evidence that the use of bone grafting along with internal fixation in the treatment of intra-articular calcaneal fractures leads to better restoration of Bohler’s angle and the prevention of late collapse. Thus, in intra-articular calcaneal fractures, if the space created after open reduction is large, bone grafts may be considered as a treatment option. Also, in this study, patients with bone grafts were able to return to full weight-bearing earlier. However, the intermediate and long-term efficacy outcomes in the two groups were similar, which means that patients with intra-articular calcaneal fractures that are treated without bone grafting have functional outcomes as good as patients with such fractures that are treated with bone grafting.
